# How is music listening purpose related to stress recovery? – two preliminary studies in men and women

**DOI:** 10.3389/fpsyg.2023.1108402

**Published:** 2023-10-10

**Authors:** Yichen Song, Ricarda Mewes, Nadine Skoluda, Urs M. Nater

**Affiliations:** ^1^Department of Clinical and Health Psychology, Faculty of Psychology, University of Vienna, Vienna, Austria; ^2^University Research Platform “The Stress of Life (SOLE) – Processes and Mechanisms Underlying Everyday Life Stress”, Faculty of Psychology, University of Vienna, Vienna, Austria; ^3^Outpatient Unit for Research, Teaching and Practice, Faculty of Psychology, University of Vienna, Vienna, Austria

**Keywords:** cortisol, music, relaxation, salivary alpha-amylase, stress recovery

## Abstract

**Introduction:**

Studies have suggested that listening to music can reduce psychological and biological responses to a stressor. However, it is unclear whether music has the same effect on stress recovery. According to field studies, people commonly use music in daily life for the specific purpose of relaxation. We explored whether individuals who generally use music for relaxation purposes show improved recovery from an acute stressor.

**Methods:**

In two independent studies, twenty-seven healthy female participants (*M*_age_ = 24.07) (Study 1) and twenty-one healthy male participants (*M*_age_ = 23.52) (Study 2) were separated into two groups based on their frequency of using music for relaxation purposes (low vs. high). All participants underwent a lab-based psychosocial stress test. Subjective stress levels were measured using visual analogue scales. Salivary cortisol and salivary alpha-amylase were measured to assess endocrine and autonomic stress responses, respectively. Subjective stress levels and saliva samples were measured nine times throughout the stress induction and recovery procedure. Chronic stress levels were assessed using the Perceived Stress Scale and the Screening Scale of Chronic Stress.

**Results:**

No significant differences were observed in subjective stress levels, salivary alpha-amylase activity, or cortisol concentration between the two groups in either of the two studies. Further analyses revealed that among male participants, increased use of music for relaxation purposes was related to more chronic stress levels (*t* (10.46) = 2.45, *p* = 0.03, *r* = 0.60), whereas female participants exhibited a trend in the opposite direction (*t* (13.94) = −1.92, *p* = 0.07, *r* = 0.46).

**Discussion:**

Contrary to our expectations, the results indicate that habitual music listening for relaxation purposes is not associated with improved recovery from a stressor. However, due to the small sample size, future exploration is necessary to enhance the statistical power of the results of the study.

## 1. Introduction

Stress is a common phenomenon in all industrialized societies (Hassard et al., 2018). It arises when an individual perceives his/her resources as insufficient to cope with the demands of the environment (Lazarus and Folkman, 1984) and encompasses a variety of negative emotional and physiological reactions, which potentially overwhelm the individual’s capacity to deal with a stressor (McEwen and Akil, 2020). It is important to distinguish between acute stress and chronic stress (McEwen, 2004): While acute stress is ubiquitous and considered an adaptive process to help individuals survive in a complex environment (McEwen and Akil, 2020), chronic stress has been shown to threaten both physical and mental health (McEwen, 1998).

As a complex process resulting from an organism being challenged by potentially threatening stimuli, stress consists of an immediate stress “reactivity” as well as a “recovery” phase, which starts after the cessation of the stressful stimulus. Stress recovery is a process that helps the individual to recover from the loss of equilibrium or balance, to regain homeostasis, and to readjust to the environment after exposure to stress (Ulrich et al., 1991; de la Torre-Luque et al., 2017b). This process is complementary to the stress response and shares the same biochemical basis. The stress process is regulated by two major systems, the hypothalamic–pituitary–adrenal (HPA) axis and the autonomic nervous system (ANS). The HPA axis exerts its effects through endocrine signals, and the most commonly used indicator of HPA activity is cortisol. Due to the quick and non-invasive nature of saliva collection, salivary cortisol is nowadays widely used as a biomarker in stress studies (Strahler et al., 2017). The ANS includes both the sympathetic nervous system (SNS) and the parasympathetic nervous system (PNS), and salivary alpha-amylase (sAA) has been increasingly used as an index for ANS activity in recent years (Nater and Rohleder, 2009).

Various methods have been explored to prevent or mitigate the harmful effects of stress on health. However, the focus has primarily been on stress reduction rather than stress recovery. Among these, music listening was widely used as an intervention due to its cost-efficiency, non-invasiveness, easy deployment, and lack of side effects (Wuttke-Linnemann et al., 2020). Both laboratory and field studies have revealed beneficial effects of music in terms of reducing stress (Thoma et al., 2013; Linnemann et al., 2015, 2016, 2018), but research on music with relation to stress recovery is scarce.

In the first study to examine an effect of music on stress recovery, Khalfa et al. (2003) observed that music listening after an acute laboratory stressor reduced cortisol levels more rapidly compared to a silence condition. Later studies also reported a positive impact of music on cardiovascular processes (de la Torre-Luque et al., 2017a,b) and improved mood (Radstaak et al., 2014; Koelsch et al., 2016) during the recovery phase. There is currently no unified operational definition of stress recovery. Khalfa et al. (2003) compared the cortisol concentration levels at different measurement points after the stress test in different groups separately. This method is able to depict the trajectory in each group after the stress test, but it failed to compare the overall differences among all measurements and between groups. Thoma et al. (2013) defined the difference between the peak values after the stressor and the first baseline value after the stressor as recovery. Other studies have conducted between-group analyzes of variance comparisons, but due to the limited number of measurement points, they were only able to compare intergroup differences in stress levels after the stress test, failing to provide an overall description of the trajectory during the stress recovery phase (Labbé et al., 2007; Groarke and Hogan, 2019; Groarke et al., 2020). In a study by Koelsch et al. (2016), repeated measures MANOVA was used with time points as a within-subject factor and stimulus conditions as between-subject factors. They reported generally higher cortisol levels in the music listening group during the recovery phase, suggesting a poorer recovery from stress compared to the control group. This method tested the overall differences between groups over time. In our current research, we decided to use the method from Koelsch et al. (2016) to test the general differences in stress levels between different groups throughout the stress induction and stress recovery phases over time.

Performing an activity with the specific purpose of improving stress recovery can be an important factor in the effectiveness of stress management interventions. According to three-pronged model of habit proposed by Wood and Rünger (2016), music listening for relaxation purpose can potentially influence stress recovery through the formation of music listening habits, even in the absence of actual music listening behavior. In line with this model, individuals who frequently engage in music listening as a means of relaxation and recovery may develop a habit where stress itself acts as a contextual cue that triggers the desire to listen to music for the purpose of recovery. Once the habit is formed, this contextual cue automatically activates the corresponding mental representation of the habit, which encompasses various sensory and perceptual features. Mentally simulating the music in this way may elicit a similar recovery effect as actually listening to the music. Besides, it is suggested that relaxing music is characterized by positive valence and low arousal (Sandstrom and Russo, 2010), including calming melodies and evoking positive emotions. If individuals mentally activate such music in response to the contextual cue, they may experience benefits in stress recovery. Additionally, the model suggests that by observing their own habitual responses, individuals are likely to infer their underlying goals. Consequently, when individuals who habitually listen to music for relaxation purpose realize that they have such music playing in their minds, it may strengthen their motivation to recover, thereby aiding in their stress recovery process. Therefore, when assessing the possible influence of music listening on stress recovery, the purpose for listening to music may be an important moderating factor.

Qualitative and quantitative studies have explored the purposes of music listening. Greasley and Lamont (2011) found that listening to music for pleasure and for relaxation were the most frequent reasons that participants reported in their daily life. In a diary study conducted by van Goethem and Sloboda (2011), the affect regulation function of music was investigated. Results revealed that individuals employed music for a range of purposes, encompassing relaxation, distraction, active coping, introspection, venting, and rational thinking. Among these purposes, relaxation was found to be the most reported. Juslin et al. (2008) found that the music listening purposes were related to specific emotional states. For example, listening to music with the purpose to relax was related to calm-contentment emotion, while the purpose to influence their feelings was related to sadness-melancholy emotion. The paper of Linnemann et al. (2015) was the first evidence that the stress-reducing effect of music listening was modulated by the purpose of music listening. They tracked the stress levels of a group of university students in daily life. Students were asked to report their stress levels and music listening behavior four times a day for 2 weeks. The results showed that music listening was related to lower subjective stress levels. At the same time, when the participants had listened to music for relaxation purposes, they showed lower subjective stress levels and lower cortisol concentrations compared to listening to music for other purposes. The findings imply that the purpose of relaxation may have a significant role in the stress management process compared to other purposes. However, this field study lacked strict control of potentially confounding variables. For instance, the study relied on retrospective assessment of stress prior to the measurements, without actively inducing a standardized stressor. This lack of controlled stress induction makes it challenging to regulate the level of evoked stress and capture the stress recovery phase. Furthermore, in daily life, there are various distractions that may influence the stress recovery process, potentially attenuating the influence of music and music listening purposes. Additionally, when collecting biomarkers such as cortisol and sAA, physical activities can significantly impact their levels. Given that physical activities are unavoidable in daily life settings, these biomarkers may not accurately reflect stress levels. These issues can be addressed through the implementation of a controlled laboratory environment. Based on the positive relationships observed in the ambulatory assessment study between listening to music for relaxation purposes and both subjective and biological stress outcomes we assume that habitual music listening for relaxation purpose helps train subjective and biological stress recovery and forms individuals’ behavior modes. The benefits of music listening may even extend to situations where individuals are unable to listen to music. Therefore, we hypothesized that individuals who habitually listen to music for relaxation may find it easier to recover from stress compared to those who do not.

As we identified a lack of studies that tested whether habitual music listening for relaxation purposes might benefit stress recovery, we analyzed existing data from two separate projects, which had different research aims to the present work. These projects implemented lab-based settings to control for potentially confounding variables. Both subjective and biological stress responses were measured, and we explored how habitual purpose of music listening was related to stress recovery. Based on previous findings, we hypothesized that habitually listening to music for relaxation purposes would be positively related to stress recovery. More specifically, we assumed that the more frequently individuals listen to music for relaxation purposes, the stronger their stress recovery will be, as measured by subjective stress levels as well as cortisol and sAA levels. Furthermore, we examined whether music listening for relaxation purposes was related to chronic stress levels.

## 2. Study 1

The data of Study 1 stem from a large project exploring the comprehensive effects of music on laboratory-induced stress (data not published yet). The project was approved by the ethics committee of the University of Vienna (reference number 00508). All of the participants provided informed consent before taking part in the experiment.

### 2.1. Materials and methods

#### 2.1.1. Participants

Previous research has indicated gender differences in HPA axis responses to stress (Kirschbaum et al., 1999) and music effects on stress-related systems (Nater et al., 2006), with women responding to both stress and music in a more sensitive manner. Therefore, this study only included female participants. The inclusion criteria were female sex, body mass index (BMI) between 18 and 25 kg/m^2^, age between 20 and 30 years, sufficient German-language ability, a regular menstrual cycle, and no pregnancy or breast-feeding. The tests were scheduled during the follicular period of participants’ menstrual cycle. To control for potentially confounding factors, the following exclusion criteria were applied: self-reported or diagnosed stress-related mental disorders; other diagnosed somatic disorders known to affect either the HPA axis or ANS; use of hormonal contraceptives, psychoactive substances or excessive consumption of alcohol or tobacco that might affect the HPA axis or ANS; being a professional or amateur-level musician; regularly practicing relaxation or mindfulness methods; hearing deficits. The inclusion and exclusion criteria were screened in a telephone interview prior to the lab appointment.

The larger project encompassed four conditions, which were researcher-selected music condition, participant-selected music condition, relaxing nature sound condition and silent control condition. A total of 105 participants ultimately completed the experiments. Study 1 used the participants from the control condition, which contained 27 participants.

#### 2.1.2. Measures

Participants’ demographic information was collected during the telephone screening. To gather information on music listening behavior, we employed the Music Preference Questionnaire (MPQ)-R (Nater et al., 2005a). To assess the participants’ use of music for relaxation purposes, participants were asked to rate the item “How frequently do you listen to music in order to relax?” on a 5-point Likert scale (1 = “never” to 5 = “very often”). None of the participants rated this item with a score of 1 (i.e., never listening to music for relaxation purposes). The remaining ratings were as follows: four participants rated 2, four rated 3, seven rated 4, and 12 participants provided a rating of 5 (i.e., listening to music for relaxation purposes very often). Given that no participant indicated never listening to music for relaxation purposes, and the sample size for each group was too small to conduct analyzes of variance (ANOVAs), two groups were formed for the subsequent t-tests: Participants who rated this item with 2 or 3 were allocated to the low-frequency group (*n* = 8) and participants were rated it with 4 or 5 were allocated to the high-frequency group (*n* = 19).

In the current study, both subjective stress levels and biological markers of stress were analyzed as dependent variables. Salivary cortisol concentration and sAA activity were analyzed from saliva samples, reflecting HPA axis and ANS activity, respectively. Saliva was collected in SaliCaps^©^ (IBL-Tecan, Hamburg, Germany) using the passive drool method. Participants were asked not to speak or swallow for 2 minutes after completing an active swallow and to subsequently transfer the saliva collected in the mouth into a tube using a straw. After collection, saliva samples were stored in a freezer (−30°C) until biochemical analysis. Subjective stress levels were measured on a visual analog scale (VAS), with participants asked at each time point to rate “How stressed do you feel at this moment?” on a line from 0 to 100.

To control for the potential impact of chronic stress, participants also completed the German version of the Perceived Stress Scale (PSS) (Klein et al., 2016) to reflect their stress levels within the last month, and the Screening Scale of Chronic Stress (SSCS) (Schulz et al., 2004) to reflect their stress levels within the last 3 months.

#### 2.1.3. Design and procedure

We used a between-subject design to compare stress recovery between female participants who frequently listen to music for relaxation purposes (high-frequency group) and those who do not (low-frequency group). Salivary cortisol, sAA, and subjective stress levels were measured to assess stress recovery after the Trier Social Stress Test (TSST) (Kirschbaum et al., 1993). The TSST has been shown to evoke a moderate level of stress in laboratory environments and to effectively activate the HPA axis and ANS (Kirschbaum et al., 1993; Nater et al., 2005b; Thoma et al., 2013). The specific procedure is described below.

Participants who met the above-mentioned criteria were invited to the lab to undergo the TSST. To control for the fluctuation of hormone levels at different time points throughout the day, the experiment was implemented in the afternoon hours, starting from 14:00. Prior to the lab appointment, participants were asked to refrain from intensive physical exercise for 24 h, from drinking alcohol or caffeinated drinks for 48 h, and from brushing their teeth, chewing gum or eating for at least an hour.

Upon arrival at the lab, participants were briefly informed about the experimental procedure and signed the informed consent form. Next, they completed the MPQ, PSS and SSCS. As the dependent variables were measured several times during the experiment, participants were also instructed on how to use VAS to report their subjective feeling of stress and how to collect saliva samples. Each participant completed nine VAS measures and saliva sample collections during the whole procedure.

**Figure 1:**
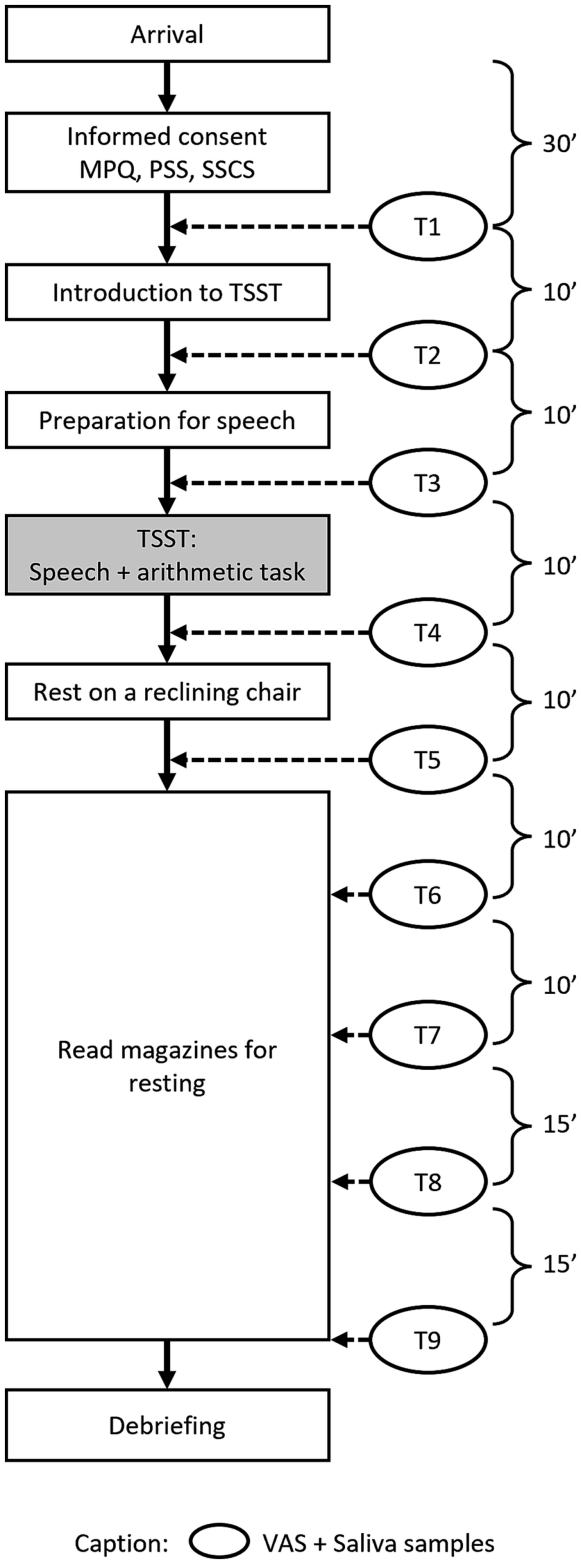
The workflow of Study 1.

Figure 1 demonstrates the workflow of study 1. After an adaptation period of 30 min, at the first time point (T1), participants provided the first saliva sample and completed a VAS. This procedure to measure stress levels was repeated at each time point throughout the experiment. Ten minutes before the TSST, participants were led into the test room, where two trained “interviewers” were seated at a table with a clearly visible video camera installed. Participants were told that they were going to undergo a mock job interview. Following these instructions, the participants completed the second stress measurement (VAS and saliva sample; T2), followed by a preparation and anticipation period of 10 min. Immediately before the TSST, stress levels were measured again (T3). The TSST paradigm consists of a speech task and a mental arithmetic task. In the present study, participants were required to give a five-minute speech for a job application and then count backwards out loud from 2,043 in increments of 17, starting again in the case of any mistakes. At the end of the arithmetic task, participants completed the fourth stress measurement (T4) and were then led back to the relaxation room, where they sat on a reclining chair for 10 min to calm down. Before getting up from the chair, the next stress measurement was conducted (T5). After this, participants were asked to sit at a desk, where they read magazines which had no emotionally arousing contents. The final four stress measurements were taken 20 min (T6), 30 min (T7), 45 min (T8), and 60 min (T9) after the TSST.

At the end of the entire experimental procedure, participants were debriefed and received monetary compensation for their participation.

#### 2.1.4. Data analysis

The data analyzes were performed using JASP software (version 0.17.1). A repeated measures mixed ANOVA was employed to examine the stress-inducing effect of TSST, stress recovery after TSST, differences between the low-and high-frequency groups, and the interaction effects. Time point was treated as a within-subject factor, while the frequency of listening to music for relaxation was considered as a between-subject factor. In the presence of significant main effects, *post hoc* tests were conducted using Bonferroni correction. T-tests were utilized to assess differences in chronic stress levels between the low-and high-frequency groups. The normality of the data was assessed using the Shapiro–Wilk test, and homogeneity of variance was examined using Levene’s test. Greenhouse–Geisser procedure was applied to correct for violations of the sphericity assumption. Statistical significance was determined at *p* ≤ 0.05. All tests were two-tailed.

### 2.2. Results

Complete data were collected from 27 participants (mean age = 24.07 years, SD = 2.25 years; mean BMI = 21.36, SD = 1.90). One participant did not provide sufficient saliva for analysis at T3. The cortisol data from this single time point were removed from the analysis. The mean PSS score was 22.44 (SD = 3.39, range = 16–29) and the mean SSCS score was 18.37 (SD = 6.73, range = 4–34). The demographic variables and chronic stress scores were compared between the high-frequency and the low-frequency group (Table 1). There were no significant differences between the groups regarding the demographic variables. The two groups did not significantly differ regarding SSCS scores or PSS scores, although the low-frequency group exhibited a trend towards higher PSS scores when compared to the high-frequency group (*t* (13.94) = −1.92, *p* = 0.07, *r* = 0.46).

**Table 1:** Sample characteristics for Study 1.

Characteristic	HF		LF		*t* (*df*)	*p*	*r*
	Mean (SD)	*n*	Mean (SD)	*n*			
Age (years)	24.00 (2.40)	19	24.25 (1.98)	8	−0.28 (15.97)	0.78	0.07
BMI	21.35 (1.97)	19	21.37 (1.85)	8	−0.02 (14.05)	0.98	0.01
PSS	21.68 (3.28)	19	24.25 (3.11)	8	−1.92 (13.94)	0.07.	0.46
SSCS	17.74 (6.20)	19	19.88 (8.10)	8	−0.67 (10.63)	0.52	0.20

SD, standard deviation; n, valid cases; HF, high-frequency group; LF, low-frequency group; BMI, body mass index; PSS, Perceived Stress Scale, SSCS, Screening Scale of Chronic Stress. *p* was calculated from two-tailed t-test; *p < 0.05.

**Figure 2:**
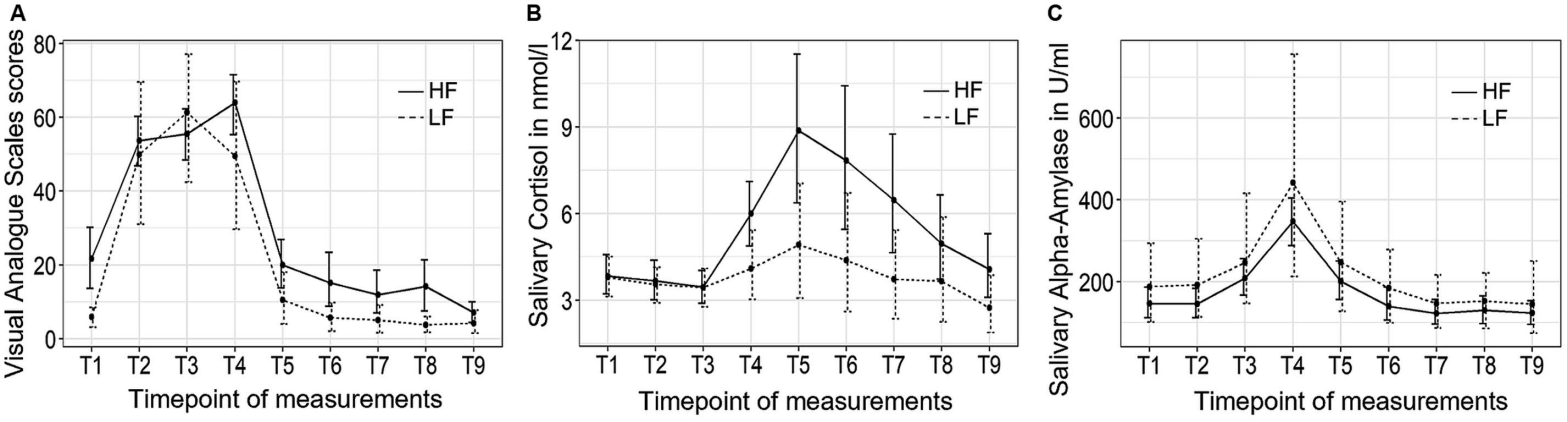
The trajectory of visual analog scale (VAS) scores **(A)**, salivary cortisol concentrations **(B)**, and salivary alpha-amylase activity **(C)** throughout the experimental procedure in Study 1. HF refers to the high-frequency group and LF refers to the low-frequency group.

Figure 2 depicts the trajectory of the three independent variables over time for the two groups. The VAS stress scores exhibited significant changes over time (*F* (3.28, 81.89) = 60.54, *p* < 0.001, *η^2^* = 0.60). However, no significant group-by-time interaction effect or group differences were observed. *Post hoc* tests revealed that the participants experienced a rise in subjective stress levels immediately after the introduction to the TSST (T2), followed by a decrease back to baseline 10 min (T5) after the conclusion of the TSST.

The salivary cortisol concentration demonstrated significant changes over time (*F* (1.32, 31.72) = 7.70, *p* < 0.01, *η^2^* = 0.10). There were no significant group-by-time interaction effect or group differences in cortisol concentration between the high-frequency group and the low-frequency group, and no group differences were found at any of the time points. Bonferroni tests revealed that the participants experienced an increase in cortisol concentration 10 min after the conclusion of the TSST (T5), and did not return to baseline level until 30 min (T7) after the completion of the TSST.

Finally, sAA activity exhibited significant changes over time (*F* (2.44, 60.98) = 23.62, *p* < 0.001, *η^2^* = 0.23). There were no significant group-by-time interaction effects or group differences observed. *Post hoc* tests revealed that sAA activity reached its peak value at the end of the TSST (T4) and returned to baseline levels 10 min (T5) after the TSST.

### 2.3. Summary of Study 1

The findings from Study 1 did not support our initial hypothesis. Contrary to our expectations, the subjective stress levels, cortisol concentration and sAA activity during the recovery phase did not significantly differ between the two groups. These findings suggest that the frequency of habitual music listening for relaxation purposes might not be related to stress recovery among female participants. Participants who listened to music less frequently for relaxation purposes exhibited higher PSS scores (reflecting the last month), though this was not statistically different.

## 3. Study 2

The data of Study 2 stem from a large project exploring the impact of group size on the effectiveness of the Trier Social Stress Test for Groups (TSST-G) (von Dawans et al., 2011) (data not published yet). The project was approved by the ethics committee of the University of Vienna (reference number 00309). All participants provided informed consent prior to the experiment.

### 3.1. Materials and methods

#### 3.1.1. Participants

The sample for Study 2 comprised men only. The inclusion criteria were age 18–35 years, a BMI of 18-28 kg/m^2^, and sufficient German-language ability. To control for confounding effects, we applied the same exclusion criteria as in Study 1.

The larger project for Study 2 included both male and female participants undergoing the TSST in group sizes of 1, 3 and 5, respectively. A convenience sample size of 20 was expected for each condition. As Study 1 only included female participants due to previous studies having reported gender differences in HPA axis responses to stress and music, we decided to complement these data with data from male participants in Study 2. Ultimately, 62 male participants completed the experiments. We used the data from the male participants who underwent the TSST in the group size of 1, consisting of 21 participants. Since we did not collect music listening-related data from female participants in Study 2, these were excluded from the analysis.

#### 3.1.2. Measures

The music listening behavior measures and stress measures were the same as in Study 1. In response to the item “How frequently do you listen to music in order to relax?,” none of the participants gave a rating of 1 (i.e., never listening to music for relaxation purposes), three participants provided a rating of 2, two participants provided a rating of 3, seven participants provided a rating of 4, and nine participants gave the maximum rating of 5 (i.e., listening to music for relaxation purposes very often). Again, participants with ratings of 2 or 3 were allocated to the low-frequency group (*n* = 5) and those with ratings of 4 or 5 were allocated to the high-frequency group (*n* = 16).

#### 3.1.3. Design and procedure

The general design and procedure of Study 2 were similar to Study 1 but with some modifications. While Study 2 also encompassed nine time points for both subjective and biological stress measures, the assignment of these time points was not exactly the same as in Study 1.

**Figure 3:**
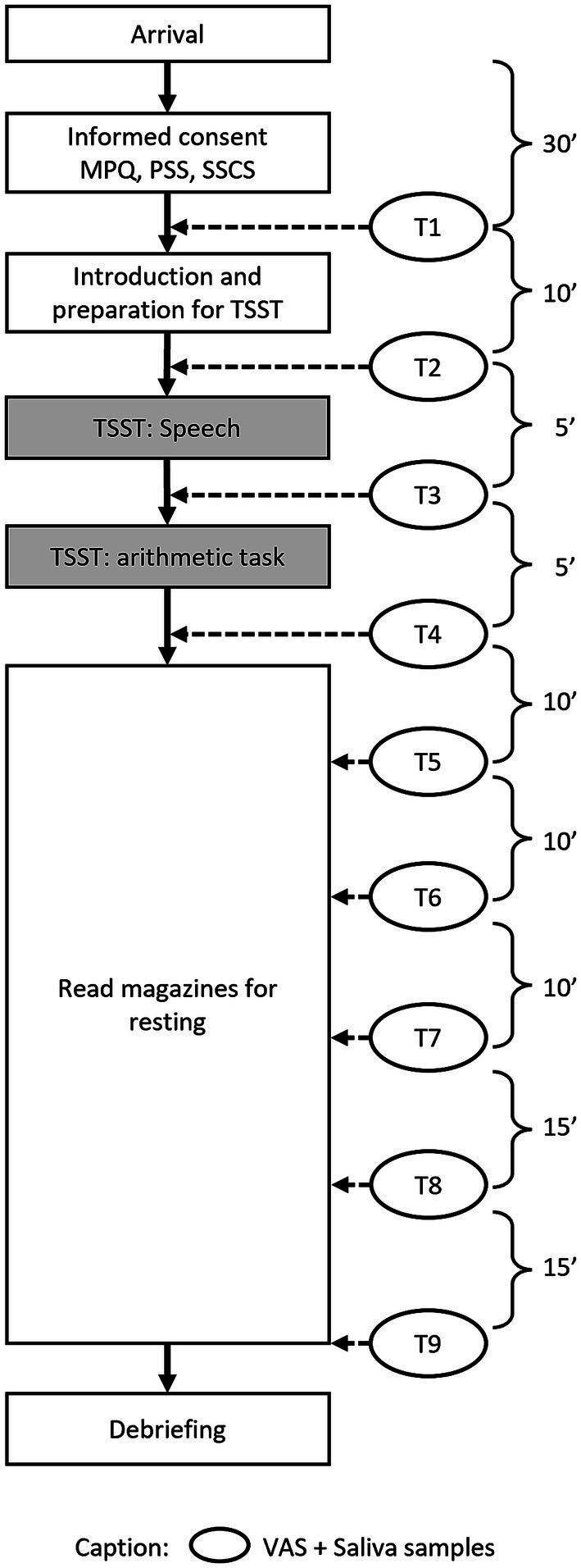
The workflow of Study 2.

Figure 3 demonstrates the workflow of study 2. Upon arrival at the lab, a 30-min adaptation period took place, followed by T1, which consisted of the first saliva sample collection and the VAS to assess baseline stress levels (T1). Next, the participants were introduced to the TSST and asked to prepare for the task. Directly before the TSST, the next stress measurement took place (T2). The third measurement occurred after the speech part of the TSST (T3) and the fourth measurement after the mental arithmetic task, which represented the end of the TSST (T4). The participants were then led back to the relaxation room, where they were provided with magazines without emotionally arousing contents and asked not to talk. During the recovery period, further stress measurements were taken 10 min (T5), 20 min (T6), 30 min (T7), 45 min (T8), and 60 min (T9) after the TSST, corresponding to Study 1.

#### 3.1.4. Data analysis

The analytical procedure was the same as in Study 1.

### 3.2. Results

Complete datasets were available from 21 participants (mean age = 23.52 years, SD = 3.71 years; mean BMI = 23.22, SD = 1.63). One participant did not complete the VAS at T5 and T6. Cortisol measurements were missing for one participant at T2 and for another participant at both T3 and T4. There were no missing data for the sAA measurements. The missing data were excluded from the analyzes. In addition, there was one missing value on the PSS and one missing value on the SSCS. The mean PSS score was 12.15 (SD = 5.93, range = 5–24) and the mean SSCS score was 13.20 (SD = 8.33, range = 2–31). The demographic variables and chronic stress scores were compared between the high-frequency and the low-frequency group (Table 2). There were no significant differences between the groups regarding the demographic variables.

**Table 2:** Sample characteristics for Study 2.

Characteristic	HF		LF		*t* (*df*)	*p*	*r*
	Mean (SD)	*n*	Mean (SD)	*n*			
Age (years)	23.81 (3.82)	16	22.60 (3.58)	5	0.65 (7.11)	0.54	0.24
BMI	23.00 (1.64)	16	23.93 (1.56)	5	−1.15 (7.04)	0.29	0.40
PSS	13.19 (6.09)	16	8.00 (2.94)	4	2.45 (10.46)	0.03*	0.60
SSCS	14.60 (8.48)	15	9.00 (6.96)	5	1.47 (8.34)	0.18	0.45

SD, standard deviation; n, valid cases; HF, high-frequency group; LF, low-frequency group; BMI, body mass index; PSS, Perceived Stress Scale, SSCS, Screening Scale of Chronic Stress. *p* was calculated from two-tailed t-test; *p < 0.05.

The high-frequency group showed significantly higher PSS scores compared to the low-frequency group (*t* (10.46) = 2.45, *p* = 0.03, *r* = 0.60). With regard to SSCS scores, there was no significant difference between the high-frequency group and the low-frequency group.

**Figure 4:**
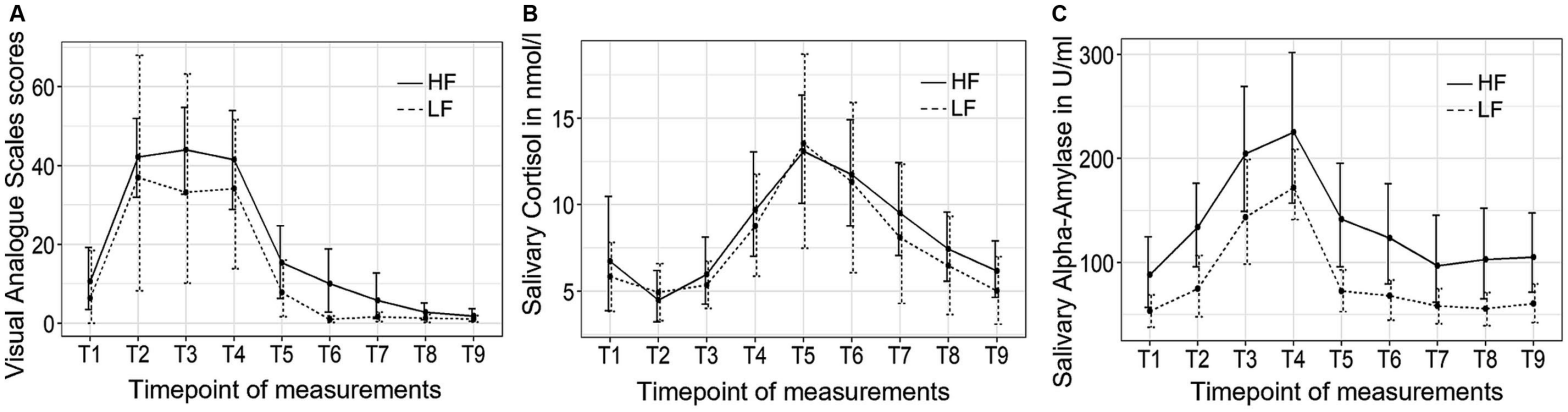
The trajectory of visual analog scale (VAS) scores **(A)**, salivary cortisol concentrations **(B)**, and salivary alpha-amylase activity **(C)** throughout the experimental procedure in Study 2. HF refers to the high-frequency group and LF refers to the low-frequency group.

Figure 4 depicts the trajectory of the three independent variables over time for both groups. The VAS stress scores exhibited significant changes over time (*F* (3.06, 55.03) = 17.28, *p* < 0.001, *η^2^* = 0.37). However, no significant group-by-time interaction effect or group differences were observed. *Post hoc* tests indicated an immediate increase in subjective stress levels following the introduction to the TSST (T2), which subsequently decreased back to baseline levels 10 min (T5) after the completion of the TSST.

The salivary cortisol concentration exhibited significant changes over time (*F* (2.25, 38.25) = 29.50, *p* < 0.001, *η^2^* = 0.27). However, no significant group-by-time interaction effect or group differences were observed. Bonferroni tests revealed that the cortisol concentration started to significantly increase at the end of the TSST (T4) and reached its peak 10 min after the TSST (T5), and did not return to baseline levels until 45 min (T8) after the completion of the TSST.

Finally, the sAA activity exhibited significant changes over time (*F* (2.49, 47.29) = 10.88, *p* < 0.001, *η^2^* = 0.13). However, no significant group-by-time interaction effect or group differences were observed. *Post hoc* tests revealed that the sAA activity increase until it reached its peak value at the end of the TSST (T4) and returned to baseline levels 10 min (T5) after the TSST.

### 3.3. Summary of Study 2

The findings of Study 2, examining male participants, likewise failed to support the hypothesis. There were no significant differences observed in subjective stress levels, cortisol concentration and sAA activity during the recovery phase between the two groups. These findings suggest that the frequency of habitual music listening for relaxation purposes might not be related to stress recovery among male participants. The frequency of listening to music for relaxation purposes was moderately related to chronic stress, insofar as higher chronic stress levels correlated with a higher frequency of listening to music for relaxation purposes for the PSS scores (reflecting the last month) but not for the SSCS scores (reflecting the last 3 months).

## 4. General discussion

The aim of the current studies was to investigate whether habitual music listening for relaxation purposes was related to better stress recovery. However, the findings were contrary to our hypothesis. Further analyzes revealed that the purpose of listening to music for relaxation showed different relations to chronic stress levels and acute stress reactions.

### 4.1. Relaxation purposes and stress recovery

Listening to music for relaxation purposes did not appear to benefit stress recovery as expected. In the current study, none of the stress measurements exhibited significant differences between the low-frequency and high-frequency groups in either of the studies. Our findings differ from the study conducted by Linnemann et al. (2015), where they found that listening to music for relaxation purposes yielded lower salivary cortisol concentrations. However, it is important to note that their study had a different experimental setup, involving actual music listening behavior. Furthermore, their focus was on stress levels rather than stress recovery. In studies specifically examining the effects of actual music listening on stress recovery, Khalfa et al. (2003) found that hearing music resulted in a cessation of cortisol increase after stress, while Koelsch et al. (2016) found that music was associated with overall higher cortisol levels compared to a control condition. These patterns diverge from our findings and suggest that the influence of habitual music listening for relaxation purpose and actual music listening on stress may differ. It is worth exploring the potential combined effects of purpose and actual music listening, which calls for further investigation.

### 4.2. Relaxation purposes and chronic/acute stress

Fancourt et al. (2014) suggested that music listening might have different effects on chronic stress and acute stress. Accordingly, the effect of the music listening purpose may also vary depending on the type of stress. To further explore the relation between relaxation purpose and stress levels, we took ongoing chronic stress into consideration. The results revealed that among male participants, an increased utilization of music for relaxation purposes was associated with higher levels of chronic stress, while female participants exhibited a nearly significant trend in the opposite direction. In the study by Linnemann et al. (2015), listening to music for the purpose of relaxation was not related to chronic stress levels. The study conducted by Linnemann et al. (2015) did not include men. In contrast, the present study conducted separate tests for male and female participants. Moreover, female participants underwent the stress test during the follicular phase, when estrogen and progesterone levels were low. The varying hormone levels could possibly explain the different recovery patterns observed between genders in the current study. However, it is important to acknowledge the limitation of a small sample size in the current study, necessitating further testing of these results among a larger group.

## 5. Limitations

While the present study is the first to specifically explore the effect of listening to music for relaxation purposes on stress recovery, several limitations need to be considered.

First, the study was a secondary analysis using data from two projects that were not designed to address the current research questions. Further studies specifically designed to investigate this topic should be conducted. Moreover, the sample sizes might have limited the range in our variables of interest. For example, in the MPQ, none of the participants reported never listening to music for relaxation purposes, and we had to form two groups based on the 5-point responses to this MPQ item. In addition, the data used in this paper came from individuals under restrictive inclusion and exclusion criteria, which limits the generalizability of the results. Furthermore, the sample consisted of young adults only. As music tastes and stress levels might vary between different age groups (Aldwin et al., 1996; LeBlanc et al., 1996), further investigations in more diverse populations are needed. Furthermore, this study solely examined the influence of the habitual listening to music for relaxation purposes on stress recovery in the absence of actual music listening. In real-life situations, it is difficult to entirely separate the act of listening to music from the purpose of obtaining relaxation. Under the circumstance of listening to music, the role of relaxation purpose on stress recovery may vary. Future studies should consider incorporating the actual music listening behavior in the investigations.

## 6. Implications

The present findings suggest that frequently listening to music for the purpose of relaxation does not substantially facilitate stress recovery. While listening to music for relaxation purposes might be helpful in a stressful situation itself, it may not support better coping with stress in situations in which music listening is not possible. Future studies might further investigate the effect of frequently listening to music for relaxation purposes on (other) coping strategies, which influence the ability to relax after a stressor. Since the present findings might partially suggest that chronically stressed persons more frequently listen to music for relaxation purposes, future studies should shed light on this possible relationship in larger samples.

The current findings may benefit clinical practice. While music has demonstrated a beneficial effect in many healthcare environments (Finn and Fancourt, 2018), this effect might not generalize to situations in which music listening is not possible. Depending on the target situation of an intervention, combining music listening with other, more generalizable coping strategies would be advisable.

## Data availability statement

The raw data supporting the conclusions of this article will be made available by the authors, without undue reservation.

## Ethics statement

The studies involving humans were approved by University of Vienna Ethics Committee. The studies were conducted in accordance with the local legislation and institutional requirements. The participants provided their written informed consent to participate in this study.

## Author contributions

All authors listed have made a substantial, direct, and intellectual contribution to the work and approved it for publication.
